# Identifying Effective Biomarkers for Accurate Pancreatic Cancer Prognosis Using Statistical Machine Learning

**DOI:** 10.3390/diagnostics13193091

**Published:** 2023-09-29

**Authors:** Rasha Abu-Khudir, Noor Hafsa, Badr E. Badr

**Affiliations:** 1 Chemistry Department, College of Science, King Faisal University, P.O. Box 380, Hofuf 31982, Al-Ahsa, Saudi Arabia; 2 Chemistry Department, Biochemistry Branch, Faculty of Science, Tanta University, Tanta 31527, Egypt; 3 Computer Science Department, College of Computer Science and Information Technology, King Faisal University, P.O. Box 400, Hofuf 31982, Al-Ahsa, Saudi Arabia; nhafsa@kfu.edu.sa; 4 Egyptian Ministry of Labor, Training and Research Department, Tanta 31512, Egypt; badrbadr539@yahoo.com; 5 Botany Department, Microbiology Unit, Faculty of Science, Tanta University, Tanta 31527, Egypt

**Keywords:** pancreatic cancer, CA19-9, CXCL-8, PCT, biomarkers, prognosis, statistical analysis, machine learning

## Abstract

Pancreatic cancer (PC) has one of the lowest survival rates among all major types of cancer. Consequently, it is one of the leading causes of mortality worldwide. Serum biomarkers historically correlate well with the early prognosis of post-surgical complications of PC. However, attempts to identify an effective biomarker panel for the successful prognosis of PC were almost non-existent in the current literature. The current study investigated the roles of various serum biomarkers including carbohydrate antigen 19-9 (CA19-9), chemokine (C-X-C motif) ligand 8 (CXCL-8), procalcitonin (PCT), and other relevant clinical data for identifying PC progression, classified into sepsis, recurrence, and other post-surgical complications, among PC patients. The most relevant biochemical and clinical markers for PC prognosis were identified using a random-forest-powered feature elimination method. Using this informative biomarker panel, the selected machine-learning (ML) classification models demonstrated highly accurate results for classifying PC patients into three complication groups on independent test data. The superiority of the combined biomarker panel (Max AUC-ROC = 100%) was further established over using CA19-9 features exclusively (Max AUC-ROC = 75%) for the task of classifying PC progression. This novel study demonstrates the effectiveness of the combined biomarker panel in successfully diagnosing PC progression and other relevant complications among Egyptian PC survivors.

## 1. Introduction

Pancreatic cancer (PC) is one of the most lethal malignant neoplasms. It is considered the seventh leading cause of cancer-related deaths worldwide [1]. The two major histological subtypes of pancreatic cancer are pancreatic ductal adenocarcinoma (PDAC), which accounts for 90% of all cases, and pancreatic neuroendocrine neoplasm (PanNEN), which accounts for 3–5% of all cases [2]. According to GLOBOCAN 2020 estimates, PC has been ranked as the 14th most common cancer in the world, counting 495,773 new cases (2.6% of new cases of all cancers combined) and causing 466,003 deaths (4.7% of all deaths caused by cancer) in 2020, with a second lower average age-standardized rate (ASR) incidence in Africa [1]. In Egypt, PC accounts for 2% of all cancers, with an age-adjusted incidence of 3.2 per 100,000, which is roughly half of the US incidence rate [3]. It was recently revealed that PC incidence rates in Egypt were much greater in urban than rural regions and that they varied greatly by district [4].

In fact, both the incidence and mortality of PC are estimated to increase over the period of 2025–2040, rates of which in Africa will be the highest in the world [5,6]. Such a poor prognosis of PC is attributed to late diagnosis, where about 80–90% of the patients have unresectable tumors at presentation [5].

For the diagnosis and prognosis of cancer patients, biological markers (biomarkers) can be reliably measured and assessed as an indicator of normal biological or pathological processes, or of pharmacological reactions to therapeutic interventions. Biomarkers are also crucial for the prediction, diagnosis, and prognosis of PC [7]. Major biomarkers of PC encompass proteomic biomarkers, including carbohydrate antigen 19-9 (CA 19-9), the only clinical PC biomarker that has been widely used in the diagnosis of PDAC and is FDA-approved [8]. In fact, CA 19-9 is the only biomarker that is recommended for clinical use by the National Comprehensive Cancer Network guidelines for PC and is considered as the most clinically useful marker for PC that reflects the tumor burden and positively correlates with the malignancy of tumor cells [9]. Moreover, changes in CA 19-9 levels among patients with locally advanced PC treated with concomitant chemoradiotherapy are thought to be a reliable prognostic marker for the survival and recurrence in patients with PC [10,11,12]. In addition, CA19-9 has been demonstrated to be a predictive biomarker both preoperatively and longitudinally following resection, as well as a predictor of resectability [13,14]. Nevertheless, CA 19-9 is not considered an individual screening tool for asymptomatic patients due to its very low positive predictive value (PPV) [15,16]. In addition to CA 19-9, other carbohydrate antigens, including CA 50, CA 72-4, CA 125, CA 195, CA 242, and carcinoembryonic antigen (CEA), have also been extensively assessed. However, they are less sensitive than CA 19-9 [17,18].

In PDAC, chronic inflammation plays a significant role in the initiation of tumors, as well as their progression and spread [19,20]. In both localized and metastatic PDAC, a systemic inflammatory response with circulating monocytes, elevated neutrophil to lymphocyte ratios, and elevated cytokines and CRP levels can be found [20]. Furthermore, the PDAC tumor microenvironment is enriched, in a complex manner, with an extracellular matrix, suppressive immune cells, fibroblasts, and soluble proteins including cytokines and growth factors [21,22,23]. In addition, Interleukin-1 (IL-1), IL-6, the chemokine (C-X-C motif) ligand 8 (CXCL-8), known as IL-8, and tumor necrosis factor-alpha (TNF-α), among other cytokines, have been linked to the diagnosis, progression, and prediction of PDAC [24,25,26,27]. Notably, it has been shown that the serum CXCL-8 level seemed to be a more accurate diagnostic marker for PC compared to classic tumor markers like CA 19-9 or CEA [28]. Previously, it has been reported that changes in postoperative serum levels of selected angiogenic cytokines, including CXCL-8, correlated with PC patients’ prognosis after resection [29]. Moreover, it has been reported that plasma levels of the cytokines monocyte chemoattractant protein-1 (MCP-1) and IL-8 correlated with decreased survival in PDAC patients who underwent partial pancreatectomy [30]. Recently, the role of selected CXC chemokines, including CXCL-8, in PDAC has been identified. The findings suggested that CXC chemokines might be used as prognostic indicators and therapeutic targets in PDAC [31,32].

Sepsis is defined as a fatal heterogeneous clinical syndrome resulting from a dysregulated host’s immune response to infection, which leads to life-threatening organ failure. It involves changes in the function of numerous body organs rather than just being a process of systemic inflammatory response or immunological disorder [33,34]. Globally, sepsis is considered as the main cause of in-hospital death [35]. Hence, sepsis has recently been recognized by the World Health Organization (WHO) as a worldwide health priority due to its high mortality and morbidity, which markedly increased upon the development of septic shock [36,37]. The mechanisms underlying the development of sepsis are extremely complex, encompassing dysregulated inflammatory response, immune dysfunction, mitochondrial failure, and coagulopathy. In addition, abnormalities in the neuroendocrine immune network, endoplasmic reticulum (ER) stress, autophagy, and other pathophysiological processes are involved, eventually resulting in organ dysfunction [38,39].

Pathogenic sepsis is not a monolithic condition, where bacterial, viral, and fungal types of sepsis do exist. In spite of the symptoms’ similarity, each pathogenic type of sepsis has its own mechanism of action. Furthermore, the etiologies and the underlying immune mechanisms exhibit sufficient differences. In fact, bacterial infections remain the primary cause of pathogenic sepsis. However, viruses and fungi still comprise a meaningful percentage of sepsis etiologies, especially among immunocompromised patients and those with other comorbidities [40].

Among the factors that primarily affect the epidemiology of sepsis is the rising prevalence of comorbidities, including cancer, its treatment, and the resulting immunosuppression. Sepsis is a common complication in immunosuppressed cancer patients [41]. Hence, its risk among cancer patients is more than ten times higher than that of the general population, with some variation depending on the type of cancer. With more than 14,000 cases per 100,000 patients, PC exhibits the greatest risk of sepsis among all other cancer patients [42]. Moreover, microbial sepsis is one of the most common causes of mortality in patients admitted to intensive care units (ICUs) worldwide [43,44]. Intensive care unit mortality rates in septic patients were approximately 26%, whereas they reached around 35% in patients with septic shock [45].

Owing to the heterogeneous nature of sepsis, the achievement of an accurate diagnosis is still challenging. Current clinical diagnostics that rely on the identification of infection-causing pathogens are misleading and relatively labor-intensive [46,47,48]. On the contrary, advanced technologies used to assess host response to infection, as well as novel individual biomarkers, result in the rapid diagnosis of sepsis [49,50,51,52]. Indeed, recent developments in timely host response diagnostics and prognostics show promise for improved outcomes. Among the established diagnostic biomarkers, procalcitonin (PCT), the precursor of the hormone calcitonin, is a reliable biomarker approved by the US Food and Drug Administration (FDA), aiding in the diagnosis of bacterial sepsis. According to several studies, PCT, alone or when combined with other clinical information, is considered an advantageous diagnostic biomarker for sepsis, as well as antibiotic therapy [53,54]. PCT exhibited greater diagnostic and prognostic value compared to the conventional biomarker, C-reactive protein (CRP). In the context of the separation of sepsis from systemic inflammatory response syndrome (SIRS), the diagnostic superiority of PCT over CRP has been reported [55,56,57]. Moreover, PCT may also function as a prognostic indicator in patients with sepsis. Even though a single PCT measurement at presentation has not shown a significant prognostic value, numerous studies have indicated that PCT can help in determining the risk of mortality when assessed serially [58,59]. Due to the complexity of sepsis, it is unlikely that a single biomarker could offer conclusive diagnosis and predictive performance. Hence, combining biomarkers that reflect various aspects of sepsis pathobiology may provide more information that is useful for effective early diagnosis and prediction. In this context, the diagnostic and/or predictive potential of a number of candidate biomarkers, including inflammatory cytokines, has been addressed, in addition to the established ones, PCT and CRP [60,61,62,63,64,65].

Machine learning (ML) is a subfield of artificial intelligence (AI) that focuses on creating algorithms with the inherent ability to identify patterns in data and gather knowledge from them in order to develop their characteristic predictions [66,67]. The implementation of both AI and its technologies, including ML, has a positive impact on cancer prevention and management as they might be utilized to investigate many aspects of cancer biology [68,69]. Accordingly, numerous studies explored the relevance of AI and ML in cancer risk assessment, diagnosis, drug development, and tumor characterization at the molecular level [70,71,72,73,74]. Moreover, ML and its subsets, including deep learning (DL), have been applied in another important aspect of cancer research, which is the prognosis/survival prediction of various types of cancers, including, among others, breast, colorectal, lung, and prostate [75,76,77,78,79,80,81].

Regarding the early diagnosis of PC, various ML algorithms have been applied to promptly locate high-risk groups via several aspects, including medical images [82,83,84], a pathological examination [85,86,87], and biomarkers [88,89,90,91]. Moreover, ML was utilized to assess the prognosis of patients with PC through the assessment of survival time [92,93,94,95], recurrence risk [96,97,98], metastasis [98,99,100], and response to therapeutic agents [101,102,103,104]. Furthermore, ML has been involved in the prediction and identification of gene mutation [105,106], nucleus segmentation detection in histopathology images [107], target localization of the tumor [108,109], and the likelihood that PC patients will be admitted to the ICU [110]. Collectively, it is evident that ML plays a significant role in so many aspects related to PC.

Pancreatic resection (PR) is among the most challenging surgical procedures that, in spite of the advancement in perioperative patient care, exhibits a high incidence of complications and is associated with significant mortality and morbidity. The occurrence of postoperative complications (POCs) is a major determinant of outcomes and its proper management is positively correlated with overall outcomes [111]. Hence, a panel of biomarkers for the early identification of patients at high risk for serious POCs/findings may help to promptly direct clinical interventions and ultimately determine the prognosis of PC patients. 

Proceeding from this, the aim of the current study was to develop a risk assessment ML-based model, based on demographic and clinical variables, as well as several accessible and cost-effective biomarkers, including CA 19-9, CXCL-8, and PCT. The proposed ML model attempts to predict risk factors for POCs including sepsis/septic shock, recurrence, or death from unknown complications among PDAC patients admitted to the ICU. The plausible clinical relevance of the ML-based prediction of POCs among PDAC patients resides in its ability to direct clinical decision making and to handle postoperative events by directing their predictions to estimate and guide optimal treatment, to minimize the complications’ impact, and to shed light on relevant risk factors in forthcoming patients.

The research contributions of the current study are as follows:

(1) Several clinical and biochemical biomarkers for 30 PC patients belonging to three groups of POCs are reported.
(2) Various statistical and exploratory data analysis techniques are applied to examine and identify the most effective panel of biomarkers for the prediction of POCs among resected PDAC patients.
(3) Statistical ML models are developed and a comparative performance analysis is performed to demonstrate the combined predictive power of the panel of significant biomarkers through an improved PC prognosis accuracy.

The remaining sections of this paper are organized as follows: Section 2 includes a detailed description of the patient grouping, biochemical assays, statistical analysis, and the ML models. Subsequently, the results and discussion are presented in Section 3 and Section 4, respectively, which are followed by the limitations and future research in Section 5, and by the conclusion, highlighted in Section 6.

## 2. Materials and Methods

### 2.1. Patient Grouping

The study group comprised 30 patients (12 women and 18 men, aged between 40 and 79 years old) diagnosed with primary adenocarcinoma of the pancreas head during the period from October 2016 to December 2017 in five different Egyptian hospitals. All patients were uniformly staged, underwent pancreatic resection, and were admitted to the ICU immediately post-surgery. Patients having concurrent cancer(s) and those with missing data were excluded (n = 25). A control group of four healthy volunteers (two women and two men, aged between 40 and 65 years old) was included. The diagnosis of PC was based on computed tomography (CT) and magnetic resonance imaging (MRI). Informed consent was obtained from all patients according to the regulations of each hospital. Pre- and postoperative (24 h after ICU admission) serum samples from PC patients and controls were collected and stored at −80 °C until assayed. According to the POCs in the ICU, PC patients were divided into three groups: group A—patients suffering from sepsis or septic shock symptoms resulting from infections by the Gram-negative bacteria *Escherichia coli* (*E. coli*) or the fungus *Candida albicans* (*C. albicans*); group B—patients with recurrent PC; and group C—patients suffering from unknown complications after radiation treatment. 

Preoperative neoadjuvant therapy (NAD) followed by surgery represents the standard of care in borderline resectable (BR) and locally advanced (LA) PDAC patients [112]. Moreover, authors have suggested some clinically significant advantages of neoadjuvant chemoradiotherapy (nCRT) compared to up-front surgery in patients with BR or LA-PDAC [113]. Hence, the treatment protocol applied to PDAC patients included in the current study involved nCRT (preoperative 5-FU-based chemoradiation) followed by resection and subsequent adjuvant radiotherapy (RT): patients were treated with intensity-modulated radiation therapy (IMRT; 31–41 Gy) combined with concurrent 5-fluorouracil (5-FU) followed by external beam radiation (EBRT). The RT dose (31–41 Gy) was delivered via a split course, with a two-week break after 20 Gy.

### 2.2. Serum Samples

A sample of 10 mL of venous blood was withdrawn from each PC patient. EDTA tubes with whole blood were used for total leukocyte count (TLC) or to obtain plasma. Serum samples were immediately tested for IgG, IgA, and PCT, or stored at −20 °C. For TLC determination, blood EDTA samples were directly used. The whole blood and serum samples were obtained by standard venipuncture from patients and healthy normal controls.

### 2.3. Biochemical Assays

Total and direct bilirubin (TBIL and DBIL, respectively), alanine transaminase (ALT), and serum albumin (ALB) were assessed using a Photometer FT-2 (Rome, Italy). Assessments were performed using specific kits according to the manufacturer’s instructions (Human, Germany).

### 2.4. Total Leukocyte Count (TLC) Determination

The total leukocyte count (TLC) was determined using an improved Neubauer’s counting chamber. Blood samples were first diluted 1:20 using Turk’s solution (gentian violet in 2% glacial acetic acid) and allowed to stand for 10 min for lysis of erythrocytes. Afterward, leukocytes were enumerated in the four large corner squares of the counting chamber and TLC was estimated [114].

### 2.5. Quantitative Determination of Serum Immunoglobulins

The serum levels of IgA and IgG were estimated using the radial diffusion plate method (Biocientifica S.A., Buenos Aires, Argentina) according to the manufacturer’s instructions. In brief, serum samples (5.0 mL) obtained from each of the included patients and controls were dispensed in separate agarose wells. Plates were firmly closed and incubated at room temperature for 48 h. A sharp precipitin ring indicated the end-point of diffusion. The concentration of IgA and IgG immunoglobulins in each sample was determined by measuring the produced ring diameter using an ocular lens that was related to a reference table for each immunoglobulin. 

### 2.6. Determination of Serum Procalcitonin (PCT)

Serum levels of PCT were quantitatively measured via a sandwich enzyme immunoassay using the Procalcitonin Human ELISA kit (Cat. # RD191006200R, BioVendor, Laboratorni medicina a.s., Brno, Czech Republic) and a TECAN Spectra-III microplate reader according to the manufacturer’s instructions.

### 2.7. Analysis of Serum Tumor Markers

#### 2.7.1. Carbohydrate Antigen 19-9 (CA 19-9)

Pre- and postoperative (24 h after the ICU admission) serum CA19-9 concentrations were estimated using the Elecsys CA19-9 immunoassay kit (Roche Diagnostics GmbH, Mannheim, Germany) and a TECAN Spectra-III microplate reader according to the manufacturer’s instructions.

#### 2.7.2. CXCL-8/IL-8

Pre- and postoperative (24 h after the ICU admission) serum levels of CXCL-8 were measured using Human IL-8/CXCL8 Quantikine ELISA Kit (Cat. # D8000C, R&D Systems, Minneapolis, MN, USA) using a TECAN Spectra-III microplate reader at 450 nm according to the manufacturer’s instructions. A CXCL-8 concentration of ≤31.2 pg/mL was considered normal.

### 2.8. Statistical Analysis

The exploratory data analysis and the relevant statistical analysis were carried out using Python libraries and packages. The statistical analysis stage is described below.

#### 2.8.1. Paired Sample *t*-Test

In the current study, the parametric pairwise *t*-tests of the clinical and biochemical parameters of different patient complication groups were carried out. The significance level for all *t*-tests was set at 95%. The mean ± standard deviation (SD) of the datasets was calculated. A pairwise *t*-test was then performed to determine whether the mean of the two complication groups are different from each other. The alternative hypothesis was that the means of the two groups are similar. 

#### 2.8.2. Correlation Analysis

In the present study, we constructed a correlation matrix showing the association between every pair of parameters in which the correlation coefficient is calculated using Pearson’s method. A pairplot analysis was also performed among different feature parameters and the complication classes, which summarizes all the data points in a single plot while explaining the linear nature of the learning problem. 

#### 2.8.3. Biomarker Importance Analysis

The importance of biomarkers was assessed using a feature selection algorithm called the random feature elimination (RFE) method in a 5-fold cross-validation (CV) framework. The RFE uses an external classifier for assigning weights to features, and then recursively selects features for elimination based on the lowest importance score relative to predicting outcome. In the current study, a random forest (RF) classifier is used as an external classifier during the feature elimination stage. RF was trained on the initial set of features and the importance of each feature is obtained through the “feature_importances” attribute. According to this specific attribute of the RF model, the least relevant features were pruned from the current set of features. This procedure was recursively iterated until the optimal number of features was retained. The RFECV method from the ‘sklearn’ package in Python was used to conduct this experiment [115]. 

#### 2.8.4. Statistical ML Modeling

Statistical machine learning is performed using different algorithms. The correlation and pairplot analysis reveal the linear relationships among features to some extent. Moreover, the dataset is relatively small for modeling any complex non-linear statistical learning algorithms. Therefore, we chose simple linear classification algorithms for modeling the relationships between the predictor variables and categorical outcomes (patient complication groups). The problem is defined as a statistical learning problem in which the patient complication, which is a dependent variable, is inferred from a set of independent observations, such as demographic, clinical, and biochemical parameters. A family of statistical ML algorithms including Gaussian naïve Bayes, multi-variate logistic regression, decision tree, ridge classifier, Gaussian process classifier, and K-nearest neighbors classifier was chosen for training the learning model. These ML algorithms are capable of learning an inference function that maps between a set of inputs and outputs using a smaller set of training data, so that the learned function can be used to predict the outcomes from the given inputs. The statistical learning process of the selected ML algorithms are explained briefly in the subsequent paragraphs in light of Hastie et al. [116]:

Gaussian Naïve Bayes (GNB):

GNB is a classification technique used in statistical ML based on the probabilistic naïve Bayes approach and Gaussian distribution. It calculates the probability of the dependent variable to be classified in a specific group based on the combined predictions by each independent variable. 

Multi-variate Logistic Regression (MLR):

MLR is a statistical ML-based classification technique that learns the relationship between dependent and multiple independent variables. The probability of output is calculated from the combinations of multiple variables. 

Decision Tree (DT):

The DT is a classification technique that relies on internal decision-making logic to learn the partitioning of the features on the basis of their values at different levels of the tree. The outcome is represented at the leaf node of the decision tree. The decision about the outcome for each sample is simply made based on its feature values. 

Ridge Classifier (RC):

The RC is a classification technique that uses Ridge regression framework and the concept of regularization. During the regularization, a penalty term is added to the cost function of the model. The penalty term forces the coefficients of the input variables to be smaller to avoid the overfitting of the model.

Gaussian Process Classifier (GPC):

GPC is a classification technique that uses probabilistic models. It is based on the generalized Gaussian process that calculates the posterior probability of the output labels by modeling the relationships between the input variables and the output labels. Additionally, the classifier reports the uncertainty of outcome predictions. 

K-Nearest Neighbor (KNN):

The KNN is a non-parametric classification technique. It uses the concept of ‘feature similarity’ to classify a new sample. The new sample will be classified into a group if it closely resembles the training samples in a neighborhood. The K-value in the KNNR model indicates the number of samples in a specific neighborhood. The proximity between samples is measured using different distance metrics, for instance, Euclidean and Manhattan distance. 

The pancreatic cancer patient dataset comprised thirty (30) sets of patient data, which were classified into three groups of POCs: ‘Sepsis’, ‘Recurrence’, and ‘Unknown complications’. The dataset was balanced as there are 10 patients under each POC category. We preprocessed the dataset so that it can be used as a training set for statistical model learning. The numeric feature parameters were standardized and the categorical features were one-hot encoded. The dataset is reconstructed including only the optimal number of features. The new dataset consisted of six features and the categorical outcome of patient POC groups. The dataset of 30 patients was then split into training and test sets with a ratio of 80:20. With this splitting, the training set consisted of 24 data points (eight (8) patients from each group), whereas the test data contained six (6) data points (two (2) patients from each complication group). The statistical ML models were trained using only the training data to learn the inference function. After the training, each model was evaluated on the independent test data. Various evaluation metrics were used to assess the performance of the learning models on the independent test data for the 3-class prediction problem. The selected evaluation metrics are accuracy, precision, recall, F1-score, and AUC-ROC score, which are suitable for the multi-class classification problem. Note that all five of these metrics use the classification outcomes of the model represented by four parameters, such as True Positive (*TP*), True Negative (*TN*), False Positive (*FP*), and False Negative (*FN*). In the binary class (positive vs. negative) classification setup, *TP* indicates the number of samples correctly predicted as the positive class; *TN* refers to the correctly predicted negative class samples. On the other hand, *FP* represents the number of negative class samples wrongly predicted as positive, whereas *FN* provides the number of positive class samples predicted as negative. In terms of the metrics, Accuracy represents the ratio of correct predictions over the total number of samples, Precision calculates the fraction of the correctly predicted positive classes among all positive class predictions, whereas Recall indicates the fraction of the positive classes that are correctly predicted. Finally, the F1-score measures the harmonic average of Precision and Recall values. The formulae for Accuracy, Precision, Recall, and F1-score metrics are shown in Equations (1)–(4). Additionally, the AUC-ROC score measures the model’s ability to distinguish between classes using various cutoff thresholds. In the case of all metrics, the larger the values are, the better the model’s performances are. The formulae for the four (4) evaluation metrics are provided in the following equations:

(1) $$\mathrm{Accuracy}=\frac{TP+TN}{TP+TN+FP+FN},$$

(2) $$\mathrm{Precision}=\frac{TP}{TP+FP},$$

(3) $$\mathrm{Recall}=\frac{TP}{TP+FN},$$

(4) $$F1\text{-}\mathrm{score}=2\times \frac{Precision\times Recall}{Precision+Recall},$$

**Figure 1:**
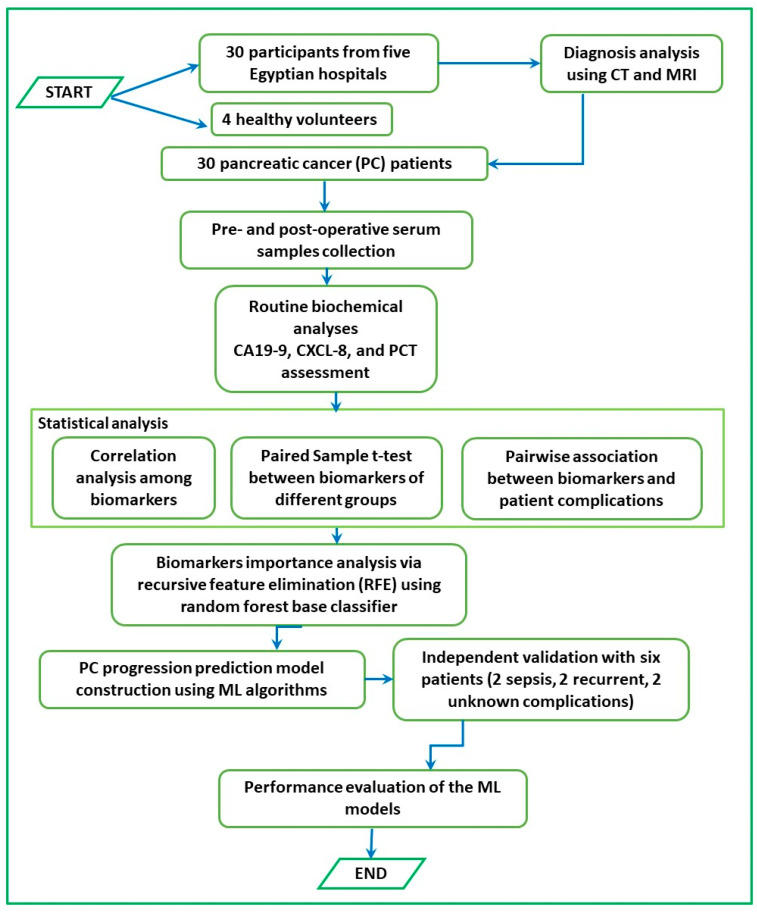
A flow chart illustrating the methodology of the current study.

The complete methodology of the current research is depicted as a flowchart in Figure 1.

## 3. Results

### 3.1. Patient Characteristics and Radiotherapy

The present study included 30 patients diagnosed with cancer in the head of the pancreas. The demographic (gender and age), treatment (dose of RT), and clinicopathological characteristics (tumor volume, cause of death, or complications developed in ICU) of all the patients are summarized in Table 1. According to the recorded POCs, the patients were classified into three groups: A, B, and C. Patients aged between 40 and 79 years and the majority of them (60%) were male. As shown in Table 1, the tumor volumeranged between 43.2 and 76.0 cc and relatively high doses of RT (31–43 Gy) were applied. It was noticed that sepsis was the main cause of death among patients in group A. About 60% of patients in group A were females, aged between 55 and 79 years old. The lowest RT dose applied was 31 Gy. The male gender, on the other hand, was the most common in group B, which represented the cancer recurrence group with the highest tumor volume and the highest RT dose of 68.1 cc and 40 Gy, respectively. In group C, 60% of patients with unknown complications were males, the youngest age was 50 years, and the highest tumor volume was 76 cc. Notably, the only survivor was a male (patient no. 10, group A). In addition, most of the dead patients were males due to unknown complications. 

### 3.2. Findings of Biochemical Assays

#### 3.2.1. Biochemical and Serological Parameters

Total bilirubin (TBIL), direct bilirubin (DBIL), alanine aminotransferase (ALT), and albumin (ALB) were assessed in the sera of PC patients and healthy controls (Table 2). Compared to healthy controls, a significant increase in levels of TBIL and DBIL was observed in patients of groups A, B, and C before surgical operation and 24 h after ICU admission (Table 2). Regarding serum ALT levels, a highly significant increase (*p* ≤ 0.05) was observed among PC patients before resection, while a significant postoperative (24 h after ICU admission) increase (*p* ≤ 0.05) was observed among patients of groups A and B. On the other hand, a significant decrease in the pre- and postoperative levels of ALB was observed among patients of groups A, B, and C, as shown in Table 2.

#### 3.2.2. Estimation of Total Leukocyte Count (TLC), Levels of IgG and IgA, and Procalcitonin (PCT)

As shown in Table 2, a non-significant difference in TLC was observed among PC patients before surgical operation compared to healthy controls. However, a highly significant increase (*p* ≤ 0.05) in postoperative TLC was observed among patients in the septic group (group A), while a significant decrease was observed among patients of groups B and C. Compared to healthy controls, a highly significant decrease (*p* ≤ 0.05) in the postoperative levels of IgG was observed among patients who suffered from sepsis (group A). However, a non-significant increase in IgG levels was noticed in those who suffered from recurrence (group B). Regarding the pre- and postoperative levels of IgA, a highly significant decrease (*p* ≤ 0.05) was observed among all patients compared to healthy controls. On the other hand, a highly significant increase (*p* ≤ 0.05) in postoperative serum PCT concentrations was observed in patients who suffered from sepsis (group A), compared to healthy controls. Nevertheless, a significant increase in PCT levels was observed among recurrent patients (group B) and patients with unknown complications (group C) as indicated in Table 2.

### 3.3. Analysis of Serum Tumor Markers

#### 3.3.1. Carbohydrate Antigen 19-9 (CA 19-9)

The pre- and postoperative serum levels of CA 19-9 were evaluated in PC patients in comparison to healthy controls (Table 3). A highly significant increase (*p* ≤ 0.05) in pre- (153.8 ± 13.9 U/mL) and postoperative (24.3 ± 1.2 U/mL) mean CA 19-9 levels were observed in patients suffering from sepsis (group A) compared to the control group. A highly significant increase in pre- (748.5 ± 59.4 U/mL) and postoperative (233.7 ± 17.22 U/mL) serum levels of CA 19-9 was observed among group B patients in comparison to the control group. On the contrary, patients in group C had the lowest pre- (52.2 ± 5.9) and postoperative (5.39 ± 0.58) CA 19-9 levels. Collectively, a 9.3-, 48.0-, and 3.3-fold increase in preoperative CA 19-9 levels were observed among patients of groups A, B, and C, respectively, compared to healthy controls. On the other hand, a marked decrease in postoperative CA 19-9 levels was observed, where there was a 1.60- (group A), 15.0- (group B), and 0.3- (group C) fold decrease compared to the control group. 

#### 3.3.2. CXCL-8/IL-8

The levels of CXCL-8 were assessed in PC patients (Table 3). A highly significant increase in pre- (107.5 ± 11 pg/mL) and postoperative (333.6 ± 29 pg/mL) mean serum levels of CXCL-8 in patients of group A was observed compared to healthy controls (22.07 ± 1.9 pg/mL). This represents a 4.9- and 15.1-fold increase in pre- and postoperative CXCL-8 levels, respectively. A comparable highly significant increase (*p* ≤ 0.05) in pre- (659.5 ± 55.4 pg/mL) and postoperative (427.1 ± 34.4 pg/mL) mean CXCL-8 levels was recorded in group B when compared to normal controls. On the other hand, a significant increase (*p* ≤ 0.05) in the postoperative mean CXCL-8 serum levels (61.6 ± 4.18 pg/mL) was observed in group C compared to the preoperative (39.95 ± 2.8 pg/mL) and normal mean levels (22.07 ± 1.9 pg/mL). Overall, the postoperative mean concentrations of CXCL-8 in groups A and C of PC patients were significantly increased compared to the preoperative ones, while a significant decrease was observed among patients of group B. Remarkably, the lowest postoperative level of CXCL-8 (26.5 ± 2.0 pg/mL) was observed in one of the patients of group A (patient no. 10) who lived for more than 180 days (Table 3). 

### 3.4. Statistical Analysis and Modeling

#### 3.4.1. Paired Sample *t*-Test of Significant Biomarkers

All pairwise parametric *t*-tests were performed among all biochemical biomarkers to investigate statistically significant differences between the markers that belong to different POC groups. The selected parametric *t*-test results are listed in Table 4. The larger the t-value, the stronger the evidence against the null hypothesis. Hence, we only reported the *t*-test results with larger t-values (i.e., t-values > 1) with associated *p*-values. With a 95% confidence interval, *p*-values < 0.05 provides stronger evidence to reject the null hypothesis. Note that the null hypothesis states that there is no significant difference between the mean values of two markers under consideration.

#### 3.4.2. Correlations between Biomarkers and Patients’ Postoperative Complications

**Figure 2:**
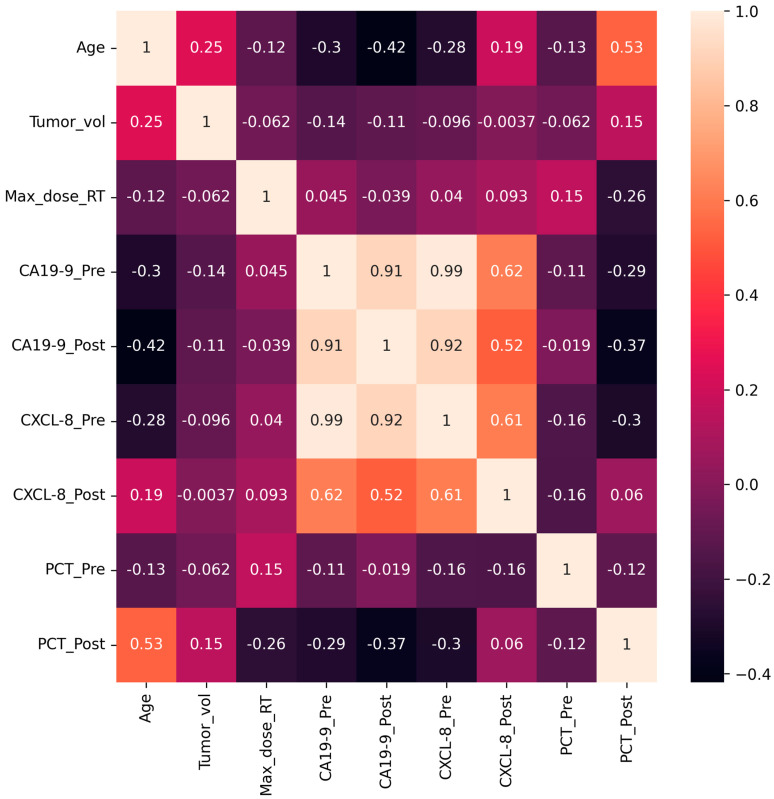
Correlation heatmap representing the correlation between all feature variables and the categorical outcome. The color palettes are explained on the right.

The heat map presented in Figure 2 shows the outcomes of the correlation coefficient analysis using Pearson’s method. Among the demographic and clinical features, the ‘Age’ and ‘Max dose of RT’ parameters had a moderate correlation (~40%) with the outcome. Among the biochemical parameters, the maximum correlation was observed for postoperative PCT, which is >80%. The only other moderately correlated features included postoperative CXCL-8 serum level with a 38% negative correlation.

The last column in the correlation matrix (Figure 2) displays the correlation of feature attributes with different POC groups. The rank of the correlated features in ascending order is as follows: age < max_dose_RT ≈ CXCL-8_post < PCT_post. Among the demographic clinical features, the age and max_dose_RT attributes exhibited higher correlations. As anticipated, the postoperative CXCL-8 and the PCT levels correlated better with the POC groups among the biomarkers. 

**Figure 3:**
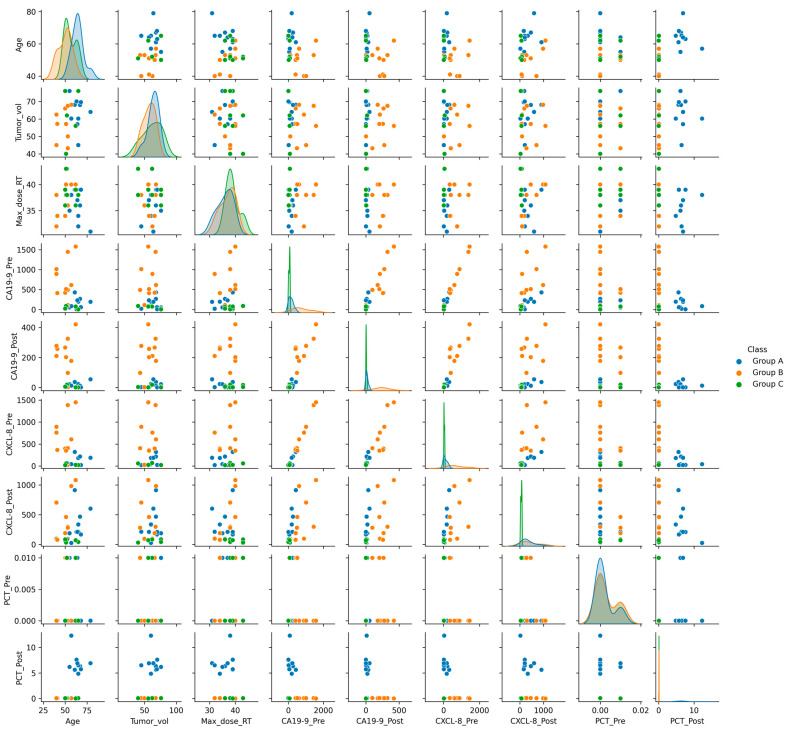
Pairplot analysis of feature parameters in the context of complication group. The blue, orange, and green colors in diagonal subplots and scatter plots indicate the three POC groups (Group A, Group B, and Group C) respectively.

The pairplot analysis of the feature variables in the context of POC groups is shown in Figure 3. The univariate distribution of each parameter is displayed as a histogram in main-diagonal subplots. The interactions between a pair of variables in the context of a patient complication group are shown as scatter plots in the upper and lower triangles. The pairplot demonstrates some linear trends in pairwise feature interactions in the context of POC groups. Particularly, the interaction between the CA19-9 and CXCL-8 biomarkers shows that we can have linear separation among data points that belong to the three POC groups. Furthermore, the analyses exhibit a few other pairwise feature interactions, for instance, CA19-9_Pre and CA19-9_Post, which can find linear classification boundaries among the three POC groups. On the other hand, other interactions (e.g., Max_Dose_RT, Tumor_vol, Age, etc.) often lead to non-linear patterns in defining the classification boundaries. 

#### 3.4.3. Identification of Important Biomarkers

**Figure 4:**
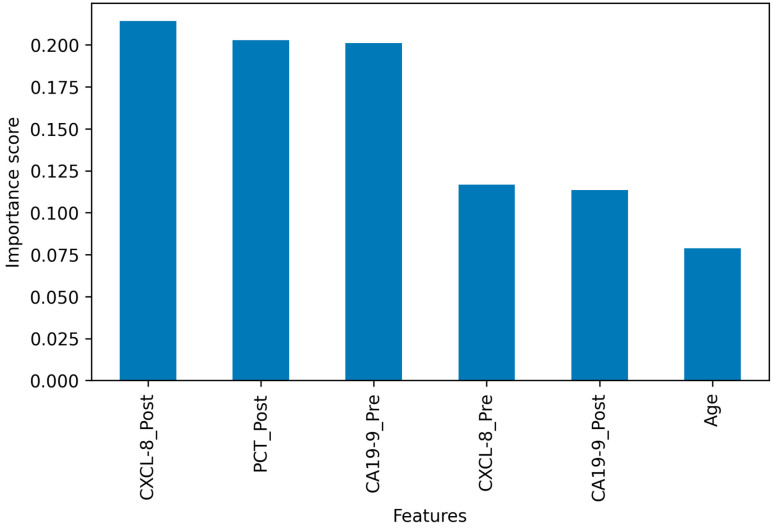
The optimal features ranked according to their importance scores as reported by the RFE CV method.

The RFE algorithm was employed to identify the optimal number of features using RF as a base classifier. The algorithm found six features to be optimal in a five-fold CV framework. Figure 4 shows the optimal features ranked by their importance scores as reported by the RF classifier. According to the feature importance scores, the CXCL-8_Post, PCT_Post, and CA19-9_Pre attributes were the top three most informative biomarkers in classifying the patients into the three POC groups. Among these three, the PCT_Post and CA19-9_Pre ones achieved similar scores of 0.20. The remaining three markers include two serum markers, CXCL-8_Pre and CA19-9_Post, and one demographic feature, age. It is important to note that ‘age’ is the only feature that was found among the top six important features, among other demographic features such as ‘gender’, as well as clinical features including ‘tumor volume’ and ‘max_dose_RT’. 

#### 3.4.4. Statistical Learning for PC Complication Prognosis

The three-class classification accuracy, precision, recall, and F1-score of the statistical learning models using the four important features on the independent test data are summarized in Table 5. All six statistical ML models show the highest performances in the case of all five evaluation metrics on the independent test set. 

The three-class classification accuracy, precision, recall, and F1-score of the statistical learning models using only CA19-9 features (CA19-9_Pre and CA19-9_Post) on the independent test data are summarized in Table 6. As shown in Table 6, the GPC, KNN, and DT classifiers were more accurate in classifying the six patients into relevant POC groups, achieving 83.33% scores in all five evaluation metrics, including Accuracy, Precision, Recall, F-score, and AUC-ROC score. The other three simpler ML models including GNB, MLR, and RC were comparatively less accurate, achieving overall scores of 66.67% in all evaluation metrics. 

## 4. Discussion

Even though only 15–20% of patients with pancreatic adenocarcinoma have resectable pancreatic cancer following thorough pre-therapeutic staging, up-front surgical resection is considered the only possible curative option [117,118]. Pancreaticoduodenectomy (PD) is commonly performed for both potentially malignant and malignant neoplasm of the pancreatic head [119]. In spite of the advancements in surgical techniques, and perioperative and critical care management, the mortality rate has dropped to less than 5%, while morbidity remains high, even in high volume centers [120,121,122]. PD is a highly invasive surgery accompanied by postoperative complications, including, among others, postoperative pancreatic fistula (POPF), delayed gastric emptying (DGE), biliary leakage, and post-pancreatectomy hemorrhage (PPH) [123,124,125]. Such complications are often accompanied by an underlying infection. Furthermore, there is a high risk of potential sepsis and mortality in the case of delayed detection or insufficient therapy [126,127]. Previous studies have reported a highly significant incidence of overall and infectious complications after PD [111,128]. Additionally, the contribution of infectious complications to complicated postoperative recovery and their association with in-hospital death were highlighted [129,130].

Sepsis is a serious clinical syndrome that is associated with high morbidity and mortality among surgical patients [131,132]. Postoperative sepsis is considered to be the major form of sepsis, as it accounts for approximately one-third of all sepsis cases [133]. It is described as a surgical complication, involving patients immediately after surgical interventions, associated with any type of infection that can lead to sepsis, severe sepsis, or septic shock [131].

Postoperative sepsis is a major health problem exhibiting an increasing incidence rate, especially in non-elective rather than elective surgeries [134]. It has been indicated that sepsis subsequent to major surgeries is common among patients admitted to the ICU [135,136]. In addition, the increased risk of postoperative sepsis following major cancer surgeries (MCSs), including pancreatectomy, has been previously reported [137]. In this regard, sepsis has been reported as one of the most frequent complications after PD [138]. Moreover, it has been demonstrated that sepsis was the only postoperative variable associated with long-term mortality among cancer patients undergoing major elective digestive surgery, including pancreatic surgery [139]. In accordance with that, one-third of the patients (n = 10) incorporated in the current study developed sepsis/septic shock following PD.

Regarding the prediction of sepsis onset, no reliable established blood markers are currently available in the clinical field. On the other hand, various established blood markers of inflammation, including PCT, are frequently altered in patients with sepsis and have been routinely used in several studies [140,141,142,143]. Nonetheless, these markers are non-specific to sepsis and are often altered in various forms of other systemic inflammation and non-septic infection. Hence, the implementation of a multi-marker approach that included clinical variables and septic biomarkers, encompassing PCT, seemed to be beneficial for the optimized management of patients with sepsis [144]. 

Recently, the usefulness of diagnostic multi-marker panels for the early detection of PDAC was established and validated using linear or non-linear classification methods that were applied for determining the optimal model [145,146,147]. Based on recent developments in the application of data-driven techniques, including ML models, in the definition, early recognition, subtype characterization, and personalization of management, we aimed to develop a comparable multi-marker approach in an attempt to aid in the prediction of sepsis and other POCs, including recurrence and other unknown complications, among PDAC patients who underwent PD. The ML models have great potential to be utilized as an alternative approach for the early prediction of sepsis, especially among ICU patients, as well as achieving a more accurate characterization and better patient care [148,149,150,151,152]. 

In this context, ML models, including random forest (RF) and naïve Bayes (NB), have been applied to identify patients from the early to peak phase of sepsis using a combination of multiple biomarkers (including PCT) and electronic medical record data (EMR; including pulse, temperature, leukocyte count, etc.). A combination of biomarkers and EMR data achieved an area under the receiver operating characteristic curve (AUROC) of 0.81 compared to an AUROC of 0.75 for EMR data alone [153]. Several retrospective studies previously demonstrated the development and validation of ML algorithms capable of the accurate prediction of sepsis onset among patients admitted to the ICU and emergency department (ED). The ML algorithms used variables that are prominent for their clinical importance in sepsis as input data, including mostly vital signs, demographic variables, and laboratory values. The applied ML algorithms surpassed tools presently used for the detection and prediction of sepsis, in which high values of AUROC, ranging from 0.68–0.99 in the ICU cohorts and 0.87–0.97 in the ED ones, have been reported [154,155,156]. Recently, a prospective clinical study implemented an ML algorithm on a wide range of patients to generate a real-time sepsis risk score. Notably, patients’ outcomes were greatly improved among septic patients who were identified early [157]. The aforementioned studies showed that ML is a potent technique capable of converting substantial quantities of electronic health records (EHR) data into prognostic models with applications in clinical decision support, resource management, as well as design and improvement of clinical workflows.

Regarding postoperative sepsis, the related clinical outcomes and risk factors have only seldom been the subject of studies. Previous findings revealed the role of ML models in the prediction of various POCs, including sepsis, among the patients who underwent major inpatient surgeries, such as gastrointestinal (GI) and surgical oncology [158,159,160,161]. Considering the prediction of risk factors for POCs accompanying pancreatic resection, several risk calculators have been developed that mostly focused on POPF or survival [162,163]. Nevertheless, very limited risk calculators for sepsis and recurrence were developed. In this context, a previous study conducted by Merath et al. developed an ML-based algorithm that effectively predicted the patient risk of experiencing POCs after liver, pancreatic, or colorectal surgery. The implemented algorithm, decision-tree learning, showed a good predictive ability for sepsis (C-statistic or AUROC of 0.79). The variables used to develop the algorithm included a significant number of clinical and laboratory variables which are readily available in the patient’s electronic health record (EHR), such as preoperative comorbidities and perioperative clinical variables, as well as 30-day postoperative complications and mortality [164]. Moreover, multiple machine-learning models, including gradient-boosted trees (GBT), KNN, and RF, were developed, trained, and assessed for their ability to predict textbook outcomes (TOs) of patients undergoing pancreatectomy, including pancreaticoduodenectomy (n = 41, 85%) [165]. TO is a multidimensional measure that reflects the ideal pancreatic surgical outcome. It is defined by the absence of postoperative pancreatic fistula, bile leak, postpancreatectomy hemorrhage, severe complications, readmission, and in-hospital mortality [166]. Accordingly, it was demonstrated that the predictive power of the implemented ML models was significantly improved upon combining patient clinical characteristics with patient activity data [165].

Pancreatic cancer (PC) patients have a poor prognosis and a high rate of recurrence. Hence, the prediction of recurrence patterns following surgery is crucial due to the application of further treatment for specific recurrent events. For determining the likelihood of the postoperative recurrence of PC, clinical characteristics such CA19-9 level, tumor size, location, stage, and degree of differentiation are quite important. Indeed, various studies have demonstrated that, among recurrence parameters of PDAC, CA19-9 is independently associated with both a one-year and two-year recurrence risk [167,168,169,170,171]. Accordingly, ML models using multi-center demographic and clinical variables were developed to predict the recurrence of PC following surgery. According to the RF algorithms used in an earlier study, the major predictors for the recurrence of PC encompassed tumor size, tumor grade, TNM stage, T stage, and lymphovascular invasion. Actually, the RF algorithm showed relatively good performance (c-index = 0.68050), thus indicating its reliability to predict the most significant risk factors for the recurrence of PC following surgery [96]. Moreover, radiomics can be used to predict the likelihood of PC recurrence in conjunction with clinical characteristics. In this regard, a local recurrence model of PDAC was developed using MLR. Data input involved clinicoradiological data and CT-based radiomic features, either separately or combined. In comparison to the clinicoradiological-only risk model (AUROC of 0.533) and the radiomics-only risk model (AUROC of 0.730), a higher performance was achieved (AUROC of 0.742) using the combined model using CT-based radiomic features and postoperative CA 19-9 elevation [172]. To predict the risk of the preoperative recurrence in PDAC, Li et al. established clinical, radiomics, and radiomics–clinical models using MLR. The authors demonstrated that the radiomics–clinical model outperformed other models in predicting one-year recurrence (AUROC 0.764 for validation set) and two-year recurrence (AUROC 0.773 for validation set). Among the clinical features involved in the combined model, preoperative CA19-9 levels showed the optimal performance in predicting the one-year recurrence with an AUROC of 0.916 (95% CI, 0.860–0.955) and 0.764 (95% CI, 0.644–0.859) in the training and validation sets, respectively. In accordance with one-year recurrence, the CA19-9 level and radiomics scores outperformed other features in predicting the status of two-year recurrence with an AUROC of 0.872 (95% CI, 0.809–0.921) in the training set and an AUROC of 0.773 (95% CI, 0.654–0.866) in the validation set [173]. 

In the present study, we have included, in addition to demographic and clinical data, pre- and postoperative laboratory data relevant to PC and its POCs, namely, sepsis and recurrence. The statistical analysis of the clinical variables and biochemical markers in the context of the patient’s complication groups revealed some important trends. The parametric *t*-test statistics presented in Table 4 showed that patients in different complication groups have statistically significant differences in CA19-9, CXCL-8, and PCT biomarkers in post-surgical cases. Specifically, both sepsis and recurrence groups (groups A and B, respectively) exhibited significant variation in the CXCL-8 marker than the group of patients with unknown complications (group C). In accordance with this, earlier studies also have highlighted the prognostic role of CXCL-8 in PC patients [26,28]. On the other hand, group B patients (recurrence) had significantly different CA19-9 levels than patients with sepsis and unknown complications. Similarly, previous studies showed that, presently, CA19-9 is the only relevant indicator for PDAC postoperative monitoring [13,174,175,176,177]. Furthermore, septic patients had significant variation in the PCT levels when compared to recurrent patients and patients with unknown complications. In line with our findings, previous results have pointed to the role of postoperative PCT levels in predicting infectious complications after pancreatic surgeries [178,179,180]. In addition to statistical analyses, we used a ML-based feature selection algorithm, RFECV, to select an optimal set of features for performing the predictive modeling of POC. The REFCV algorithm, using RF as the base learner, reported a set of six features, namely, the pre- and postoperative values of CXCL-8, the pre- and postoperative values of CA19-9, and the postoperative value of PCT, and the only demographic variable ‘age’, as the most significant attributes. These features turned out to contribute the highest to learning the inference function that maps the feature attributes to one of the POC groups. 

In the current study, in the case of ML model selection, we emphasize choosing non-complex linear classification algorithms that are capable of learning from a limited number of training data points, such as GNB, MLR, RC, GPC, DT, and KNN. Although the models are trained and evaluated on a small set of patients, both the training and test sets were made balanced to ensure the learning is not biased to the majority group. Standardizing the features and using the most relevant attributes became two crucial factors that assisted the learning models in demonstrating great accuracy on unknown test data. We used a set of six optimal features, i.e., the pre- and postoperative values of CXCL-8, the pre- and postoperative values of CA19-9, and the postoperative value of PCT, and the demographic variable ‘age’, to train the selected ML models for the three-class POC classification problem. All six ML models achieved 100% accuracy with similar scores of precision, recall, and F1 scores when evaluated on independent test data. The six algorithms also produced AUC-ROC scores of 100% on the test data, which indicates that the predictive models were successfully able to distinguish between the three POC groups using various cutoff thresholds for classification. 

As CA19-9 is the most widely utilized biochemical marker for detecting the postoperative recurrence of PC, it was interesting to assess the predictive power of CA19-9 features in distinguishing between various POC groups. Toward that objective, we performed a similar three-class patient POC prediction using the pre- and postoperative CA 19-9 values (Table 6). The prediction models achieved the highest accuracy of 83.33% using the GNB, DT, and KNN classification algorithms. The confusion matrices revealed that these three learning models misclassified one of the septic patients as an unknown group patient, whereas the models with lesser accuracy (e.g., GNB, MLR, and RC) had two misclassifications for the septic POC group patients. Specifically, the models mispredicted both septic patients (group A) into either recurrent (group B) or unknown complication (group C). For the other two complication groups, the models were able to correctly classify all the patients. Accordingly, CA19-9 was not an adequate feature for any ML model to distinguish the septic patients from the patients with recurrence and unknown complications in the current dataset. On the other hand, the same ML algorithms were able to accurately identify patients of all POC groups when the combined panel of biomarkers including other serum markers such as PCT and CXCL-8 (pre- and post-surgical values), and the demographic variable ‘age’, was used as input features. This ML experiment establishes the effectiveness of combined biomarker panels in successfully predicting POCs among a population of Egyptian PC patients.

## 5. Limitations and Future Research

Admittedly, the patient sample size in the current study is small, largely due to the low incidence of PC in Egypt, which rendered the collection of cases difficult. In addition, we tried to select a homogenous sample of patients who underwent pancreatic resection, especially pancreaticoduodenectomy (PD), and all followed the same treatment protocol. However, using effective statistical analysis and suitable ML techniques, the present study was able to identify an effective panel of biomarkers for modelling the prediction of POCs in PDAC patients. The performances of the statistical learning models using these six biomarkers were evaluated on a small group of patients. In general, the statistical ML modeling is data-driven and the reliability of ML model predictions requires an investigation on moderate-size independent test data. Hence, future focus will be given to enhancing the current dataset through gathering more patients’ information. This is crucial for performing reliable and robust training and testing of the statistical learning models. Furthermore, the selected biomarkers were assessed at only two specific time points, i.e., preoperative and 24 hours after ICU admission. Additional time points are recommended to enhance the predictive capacity and effectiveness of the identified biomarkers.

Future research considering other potentially relevant biomarkers, as well as other patient characteristics (e.g., stage and Eastern Co-operative Oncology Group Scale of Performance Status (ECOG PS)), as predictive factors is indispensable. The subsequent research can aim to improve the prediction of the assessed postoperative events and others among resected PDAC patients. Evaluation with external datasets is highly recommended to assess the generalization ability of the proposed model.

## 6. Conclusions

The current study assessed and identified the ability of a panel of demographic and clinical variables as well as accessible and cost-effective biomarkers to predict POCs in 30 PC patients in Egyptian settings using the appropriate statistical analyses and ML modeling. The biomarkers that are assessed included CA19-9 and CXCL-8 (IL-8), both known to be correlated with PC patients’ diagnosis and prognosis. Moreover, the combined biomarker panels, including CA19-9 and inflammatory cytokines, inclusive of CXCL8 (IL-8), have been shown to improve the diagnostic accuracy of PDAC. The assessed biomarkers also incorporated PCT, which is one of the most commonly used biomarkers to detect postoperative complications and is considered as a powerful diagnostic biomarker for sepsis, as well as a therapeutic guide to antibiotic treatments. The statistical analyses, such as *t*-test, correlation, and pairplot analyses, followed by a feature importance study, revealed that biomarkers such as pre- and postoperative CXCL-8 and PCT are very promising biomarkers for the prediction of POCs of PD, including sepsis and recurrence, in addition to the classical tumor marker, CA19-9. Specifically, this study identified a panel of six features, namely, the pre- and postoperative CA19-9 and CXCL-8, the postoperative PCT, and the patient’s age, for the task of POCs’ prediction. This combination of biomarkers resulted in a 100% accurate prediction when employed as statistical learning features in six various ML algorithms. The present research establishes the effectiveness of the combined clinical and biochemical markers in the prediction of POCs among resected PC patients using ML techniques through independent validation and paves the way for achieving higher accuracy and precision in PC prognosis tasks. 

## Figures and Tables

**Table 1:** The demographic (gender and age), treatment (dose of radiotherapy (RT)), and clinicopathological characteristics (tumor volume, cause of death, or complications developed in ICU) of patients.

Patient No.	Gender	Age (Year)	Tumor Volume (cc)	Maximum Dose of RT (D_max_, Gy)	Cause of Death or Complications in ICU
**Group A**					
**1**	F	65	60.2	34.0	Septic shock
**2**	F	61	68.1	39.0	Sepsis
**3**	F	67	68.0	36.0	Sepsis and septic shock
**4**	F	55	76.0	35.0	Sepsis with renal and liver impairment
**5**	M	79	64.0	31.0	Septic shock
**6**	M	65	45.0	32.0	Sepsis
**7**	F	68	69.6	39.0	Sepsis and septic shock
**8**	M	63	70.0	39.0	Sepsis
**9**	F	64	57.0	37.0	Septic shock
**10**	M	57	60.2	38.0	Sepsis *
**Group B**					
**1**	F	50	66.0	34.0	Recurrence
**2**	F	40	62.5	32.0	Recurrence
**3**	M	57	68.1	40.0	Recurrence
**4**	M	53	67.5	38.0	Recurrence
**5**	M	53	43.2	38.0	Recurrence
**6**	M	41	57.2	34.0	Recurrence
**7**	M	40	45.0	38.0	Recurrence
**8**	M	53	50.0	36.0	Recurrence
**9**	M	51	57.1	40.0	Recurrence
**10**	M	62	56.0	40.0	Recurrence
**Group C**					
**1**	F	52	62.0	43.0	Unknown
**2**	M	51	40.0	38.0	Unknown
**3**	F	50	76.0	39.0	Unknown
**4**	M	65	76.0	38.0	Unknown
**5**	M	62	56.0	36.0	Unknown
**6**	F	52	62.0	38.0	Unknown
**7**	F	51	40.0	43.0	Unknown
**8**	M	50	76.0	36.0	Unknown
**9**	M	65	76.0	38.0	Unknown
**10**	M	62	56.0	39.0	Unknown

* Patient lived more than 180 days.

**Table 2:** Collective pre- and postoperative (24 h after ICU admission) biochemical (TBIL and DBIL, ALT, and ALB), immunological (TLC, IgG, and IgA), and PCT data obtained from PC patients (n = 30) and healthy controls (n = 4).

Biochemical Test	Preoperative Findings	Postoperative Findings			Healthy Controls
		Sepsis (Group A: 10)	Recurrence (Group B: 10)	Unknown Complications (Group C: 10)	
**TBIL (mg/dL)**	2.3 ± 0.5 **	0.87 ± 0.1 *	0.84 ± 0.1 *	0.86 ± 0.1 *	0.72 ± 0.1
**DBIL (mg/dL)**	0.78 ± 0.08 **	0.16 ± 0.01 *	0.15 ± 0.05 *	0.16 ± 0.05 *	0.13 ± 0.01
**ALT (U/L)**	106.0 ± 8.5 **	26.0 ± 1.2 *	21.0 ± 0.9 *	15.0 ± 0.9 *	19.0 ± 1.0
**ALB (g/L)**	4.14 ± 0.3 **	3.5 ± 0.2 **	4.35 ± 0.3 *	4.35 ± 0.3 *	4.7 ± 0.5
**TLC (×10^3^/mm^3^)**	5.99 ± 0.085 ^ns^	17.12 ± 0.15 **	4.2 ± 0.331 *	4.0 ± 0.331 *	5.8 ± 0.04
**IgG (g/L)**	8.01 ± 0.6 ^ns^	5.96 ± 0.44 **	8.76 ± 0.7 ^ns^	8.01 ± 0.7 ^ns^	8.57 ± 0.76
**IgA (×10^2^ g/L)**	9.41 ± 0.8 **	7.8 ± 0.6 **	7.81 ± 0.7 **	7.71 ± 0.7 **	31.2 ± 2.5
**PCT (ng/mL)**	0.00	6.9 ± 0.8 **	0.072 ± 0.01 *	0.070 ± 0.001 *	0.01

^ns^ = non-significant; * Changes were significant at *p* ≤ 0.05; ** Changes were highly significant at *p* ≤ 0.05.

**Table 3:** Pre- and postoperative (24 h after ICU admission) serum levels of CA19-9 (U/mL) and CXCL-8 (pg/mL) in patients diagnosed with PC (n = 30) and healthy controls (n = 4).

Patient No.	Preoperative CA19-9 (U/mL)	Postoperative CA19-9 (U/mL)	Preoperative CXCL-8 (pg/mL)	Postoperative CXCL-8 (pg/mL)
**Group A**				
**1**	196.0 ± 29.0 **	23.5 ± 2.0 **	182.0 ± 13.0 **	336.0 ± 35.0 **
**2**	429.0 ± 39.0 **	37.0 ± 2.0 **	319.0 ± 40.0 **	912.0 ± 82.0 **
**3**	267.0 ± 18.0 **	12.0 ± 1.1 **	215.0 ±17.0 **	467.0 ± 48.0 **
**4**	53.0 ± 6.0 **	22.0 ± 1.0 **	42.1 ±37.0 **	189.0 ± 17.0 **
**5**	193.0 ± 17.0 **	55.0 ± 4.0 **	187.0 ± 15.0 **	602.0 ± 55.0 **
**6**	19.8 ± 1.8 **	2.5 ± 1.0 **	20.3 ± 1.0 **	214.0 ± 22.0 **
**7**	58.0 ± 5.0 **	1.2 ± 0.1 **	22.0 ± 1.0 **	168.0 ± 15.0 **
**8**	6.47 ± 0.3 **	1.3 ± 0.1 **	24.0 ± 1.0 **	210.0 ± 17.0 **
**9**	231.0 ± 15.0 **	1.2 ± 0.1 **	22.0 ± 1.0 **	211.5 ± 17.0 **
**10**	85.0 ± 8.0 **	12.7 ± 1.0 **	42.1 ± 6.0 **	26.5 ± 2.0 **
**Mean ± SD**	**153.83 ± 13.9** **	**24.3 ± 1.2** **	**107.5 ± 11.0** **	**333.6 ± 29.0** **
**Group B**				
**1**	421.0 ± 55.0 **	266.0 ± 18.0 **	396.0 ± 36.0 **	191.0 ± 18.0 **
**2**	891.0 ± 111.0 **	211.0 ± 15.0 **	759.0 ± 81.0 **	98.0 ± 6.0 **
**3**	613.0 ± 29.0 **	178.0 ± 16.0 **	606.0 ± 50.0 **	981.0 ± 96.0 **
**4**	1444.0 ± 112.0 *	325.0 ± 26.0 **	1384.0 ± 112.0 **	298.0 ± 22.0 **
**5**	491.0 ± 40.0 **	98.0 ± 8.0 **	405.0 ± 36.0 **	281.0 ± 21.0 **
**6**	412.0 ± 36.0 **	257.0 ± 16.0 **	365.0 ± 11.0 **	79.0 ± 8.0 **
**7**	1012.0 ± 98.0 **	277.0 ± 17.0 **	891.0 ± 66.0 **	704.0 ± 60.0 **
**8**	112.0 ± 10.0 **	2.7 ± 0.1 **	26.3 ± 2.0 **	96.2 ± 6.0 **
**9**	512.0 ± 53.0 **	203.0 ± 19.0 **	349.0 ± 28.0 **	462.0 ± 12.0 **
**10**	1577.0 ± 150.0 *	421.0 ± 36.0 **	1448.0 ± 132.0 *	1081.0 ± 95.0 **
**Mean ± SD**	**748.5 ± 59.4** **	**233.7 ± 17.22** **	**659.5 ± 55.4** **	**427.1 ± 34.4** **
**Group C**				
**1**	80.0 ± 17.0 **	17.0 ± 1.5 **	65.0 ± 6.0 *	85.0 ± 7.0 *
**2**	84.0 ± 6.5 **	3.5 ± 0.5 **	56.0 ± 4.0 *	32.5 ± 3.0 *
**3**	23.6 ± 3.0 **	5.7 ± 0.2 **	31.0 ± 1.0 *	66.0 ± 2.9 *
**4**	3.34 ± 0.5 **	1.2 ± 0.6 **	25.3 ± 1.0 *	35.8 ± 1.0 *
**5**	65.7 ± 5.0 **	1.3 ± 0.1 **	29.0 ± 2.0 *	78.0 ± 7.0 *
**6**	75.0 ± 17.0 **	19.0 ± 1.5 **	69.0 ± 6.0 *	85.0 ± 7.0 *
**7**	89.0 ± 6.5 **	3.5 ± 0.5 **	59.0 ± 4.0 *	32.5 ±3.0 *
**8**	23.6 ± 3.0 **	5.7 ± 0.2 **	19.5 ± 1.0 *	86.0 ± 2.9 *
**9**	3.34 ± 0.5 **	1.2 ± 0.6 **	21.3 ± 1.0 *	42.3 ± 1.0 *
**10**	75.7 ± 5.0 **	1.3 ± 0.1 **	25.0 ± 2.0 *	72.0 ± 7.0 *
**Mean ± SD**	**52.2 ± 5.9** **	**5.39 ± 0.58** **	**39.95 ± 2.8** *	**61.6 ± 4.18** *
**Healthy controls**				
**1**	11.0 ± 1.0	-	23.0 ± 2.0	-
**2**	18.0 ± 1.0	-	21.8 ± 2.0	-
**3**	12.3 ± 1.0	-	22.0 ± 2.0	-
**4**	21.2 ± 1.2	-	21.5 ± 1.5	-
**Mean ± SD**	**15.6 ± 2.0**	**-**	**22.07 ± 1.9**	**-**

* Changes were significant at *p* ≤ 0.05; ** Changes were highly significant at *p* ≤ 0.05.

**Table 4:** Paired-sample *t*-test statistics of postoperative biochemical parameters between different POC groups. The G1, G2, and G3 in the table represent the three POC groups, the ‘Sepsis’, ‘Recurrence’, and ‘Unknown complications’ classes, respectively.

Sample Parameters		t-Value	*p*-Value	Cohen-d
**CA19-9_post**	G2 vs. G3	5.91106	0.00023	2.64
	G1 vs. G2	−5.448325	0.00041	2.48
	G1 vs. G3	1.686535	0.125969	0.804603
**CXCL-8_post**	G2 vs. G3	3.076225	0.01322	1.387486
	G1 vs. G2	−0.564692	0.58608	0.291199
	G1 vs. G3	3.236429	0.010217	1.470414
**PCT_post**	G2 vs. G3	0.631454	0.543447	0.059925
	G1 vs. G2	10.414405	0.00003	4.701717
	G1 vs. G3	10.433683	0.000003	4.702802

**Table 5:** Summary of three-class classification performance of the six statistical learning models on the independent test data using combined markers.

Model	Accuracy	Precision	Recall	F-Score	AUC-ROC Score
GNB	100	100	100	100	100
MLR	100	100	100	100	100
RC	100	100	100	100	100
GPC	100	100	100	100	100
KNN	100	100	100	100	100
DT	100	100	100	100	100

**Table 6:** Summary of three-class classification performance of the six statistical learning models on the independent test data using CA19-9 markers (CA19-9_Pre and CA19-9_Post) only.

Model	Accuracy	Precision	Recall	F-Score	AUC-ROC Score
GNB	66.67	66.67	66.67	66.67	50
MLR	66.67	66.67	66.67	66.67	50
RC	66.67	66.67	66.67	66.67	50
GPC	83.33	83.33	83.33	83.33	75
KNN	83.33	83.33	83.33	83.33	75
DT	83.33	83.33	83.33	83.33	75

## Data Availability

All data analyzed during this study are included in this published article.
